# Stability of Circulating Exosomal miRNAs in Healthy Subjects

**DOI:** 10.1038/s41598-018-28748-5

**Published:** 2018-07-09

**Authors:** David Sanz-Rubio, Inmaculada Martin-Burriel, Ana Gil, Pablo Cubero, Marta Forner, Abdelnaby Khalyfa, Jose M. Marin

**Affiliations:** 1 Translational Respiratory Research Unit, IISAragon & CIBERES, Hospital Universitario Miguel Servet, Zaragoza, Spain; 2 Laboratory of Biochemical Genetics, IIS Aragón, Universidad de Zaragoza, Zaragoza, Spain; 3 Section of Sleep Medicine, Department of Pediatrics, Pritzker School of Medicine, Biological Sciences Division, The University of Chicago, Chicago, IL USA; 4 Department of Medicine, University of Zaragoza, Zaragoza, Spain

## Abstract

Exosomes are nano-vesicles present in the circulation that are involved in cell-to-cell communication and regulation of different biological processes. MicroRNAs (miRNAs) are part of their cargo and are potential biomarkers. Methods of exosome isolation and the inter-individual and intra-individual variations in circulating miRNA exosomal cargo have been poorly investigated. This study aims for comparing two exosome isolation methods and to assess the stability of eleven plasma exosomal miRNAs over time. In addition to evaluate miRNA variability of both kits, the effect of freezing plasma before exosome isolation or freezing isolated exosomes on miRNA stability was also evaluated. MiRNA levels were tested in 7 healthy subjects who underwent four different blood extractions obtained in 4 consecutive weeks. One of the isolation kits displayed generally better amplification signals, and miRNAs from exosomes isolated after freezing the plasma had the highest levels. Intra-subject and inter-subject coefficients of variance were lower for the same isolation kit after freezing plasma. Finally, miRNAs that showed an acceptable expression level were stable across the consecutive extractions. This study shows for the first time the stability over time of miRNAs isolated from circulating plasma exosomes, establishing a key step in the use of exosomal miRNAs as biomarkers.

## Introduction

Exosomes are double-membrane vesicles with a size between 30 and 150 nm that are formed by the recruitment of the protein Alix to the surface of early endosomes^1^. Exosomes play an essential role in cell-to-cell communication and are involved in both normal physiological processes, such as immune responses^2^, and the development of diseases, including cardiovascular diseases^3^. Exosomes are secreted by many cell types, including B cells^4^, dendritic cells^5^, T cells^6^, platelets^7^ and tumor cells^8^. Exosomes have been isolated from different biofluids, including plasma^9,10^, urine^11,12^, cerebrospinal fluid^13,14^, breast milk^10^ and saliva^10^. Their widespread localization makes them and their cargo suitable for the development of new biomarkers. In addition to proteins, exosomes contain other biological molecules, such as lipids or RNA, including messenger RNA (mRNA) and microRNA. The identification of mRNAs and miRNAs in exosomes and the ability of the transferred exosomal mRNA and miRNA to be translated in target cells represent a major breakthrough in exosome biology. Although mRNAs are the most abundant, the specificity and importance of miRNAs in post-transcriptional regulation has indicated their potential as biomarkers. These diverse cargos in exosomes provide unique opportunities for biomarker discovery and the development of noninvasive diagnostic tools.

Exosomes can intercellularly transfer miRNAs, thus participating in miRNA-based signaling, and the dysregulation of miRNA activity can lead to the development of a variety of diseases^15,16^. For example, miRNAs are 18- to 25-nucleotide small RNA species, and their dysregulation has a major role in different pathologies, such as cardiovascular diseases^17^ or cancer^18–20^. MiRNAs can be secreted bound to proteins, such as Ago-2^21^ or HDL^22^, packaged in vesicles^23^ or exist freely in plasma or serum^18,19,24^. MiRNAs are loaded into the exosomes specifically through mechanisms mediated by proteins such as ubiquitin, the Endosomal Sorting Complex Required for Transport (ESCRT) and Y-box protein^25^, or miRNA motifs recognized by proteins, such as hnRNPA2B1^26^. This selective packaging explains why exosomes from diseased individuals contain miRNAs different from those found in healthy subjects^27^.

The potential of exosomal miRNAs as biomarkers for translation to the clinic has been poorly assessed. The ideal biomarker must be accurate, sensitive and specific. The first step in evaluating the capability of exosomal miRNAs to act as biomarkers is to demonstrate their stability in healthy subjects. The aim of this study was to assess the stability of a set of miRNAs involved in inflammation and cardiovascular diseases prior to its application in clinical research.

## Results

### Subjects

We included 3 males and 4 females from ages 25 to 62 years, with no age difference between genders. A summary of the clinical data of each subject is shown in Table 1. Blood samples from each patient were obtained in four visits separated by approximately one week.

**Table 1:** Clinical data of the subjects.

Subject	Gender	Age	BMI kg/m^2^	Glucose mg/dL	Triglycerides mg/dL	Cholesterol mg/dL	HDL mg/dL	LDL mg/dL
C1	Male	25	20,66	84	81	203	83	104
C2	Female	25	22,00	80	74	213	50	148
C3	Female	34	21,36	82	75	165	43	107
C4	Female	29	20,80	83	62	200	73	115
C5	Male	61	25,31	89	145	176	50	97
C6	Male	35	27,16	90	69	177	56	107
C7	Female	47	23,66	87	55	137	56	70
Mean		36.6 ± 13	23 ± 2.5	85 ± 3,7	80.1 ± 29.9	181.6 ± 14.2	58.7 ± 14.2	106.9 ± 23.2

*Abbreviations*. BMI: Body mass index, HDL: High density lipoprotein, LDL: low density lipoprotein.

### Exosome isolation and characterization

**Figure 1:**
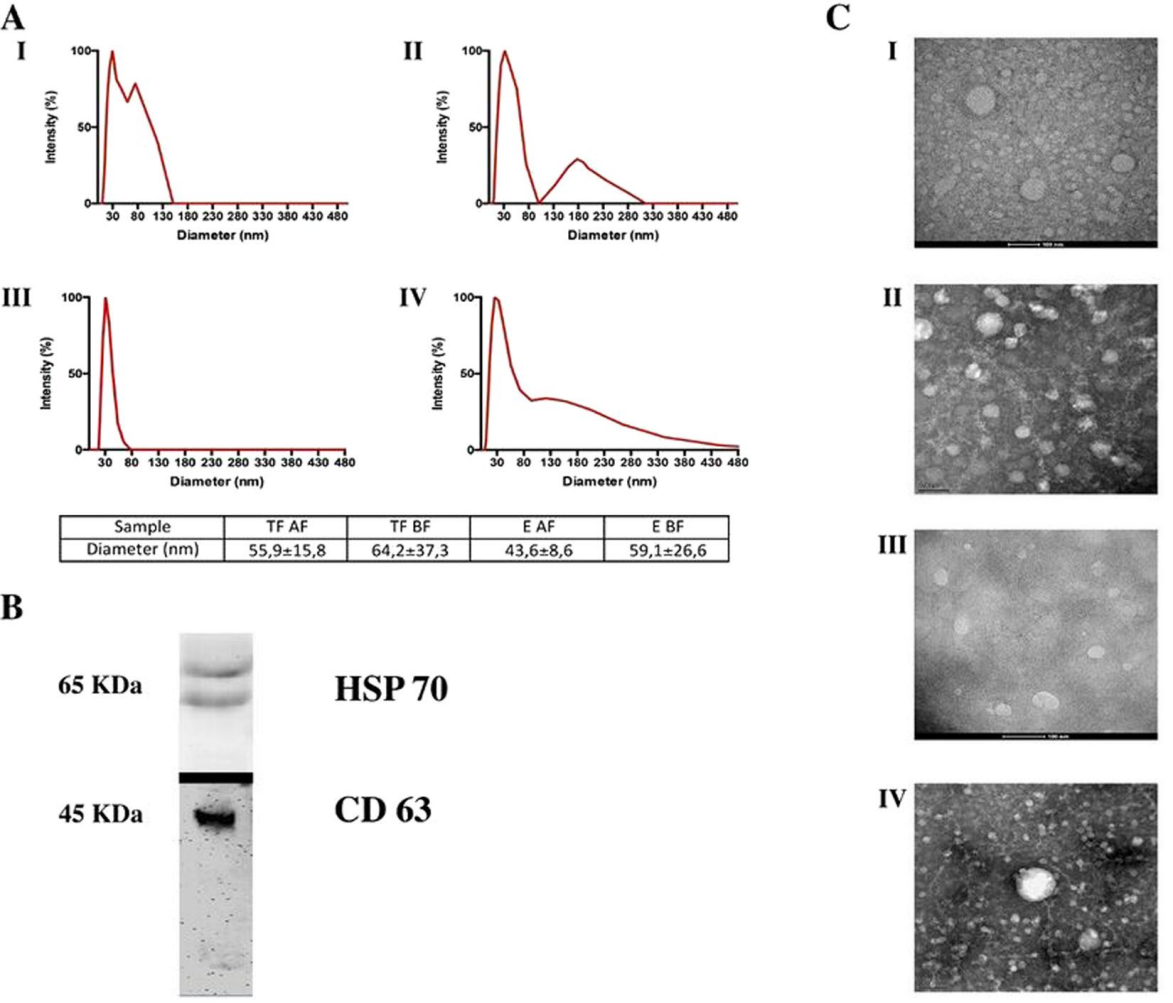
(**A**) Representative curves of DLS assays performed for the 4 isolation methods (I) TF-AF, (II) TF-BF, (III) EX-AF and (IV) EX-BF and the mean diameters obtained. (**B**) Representative Western blot bands of the exosomal proteins HSP70 and CD63 displayed on their individual Western blot assays. (**C**) Representative exosome images taken by electron microscopy (I) TF-AF, (II) TF-BF, (III) EX-AF and (IV) EX-BF.

We prioritized extraction of high quantity exosomes using two different commercial kits, the miRCURY Exosome Isolation Kit (Exiqon, EX) and Total Exosome Isolation Kit (Thermo Fisher Scientific, TF). The presence of exosomes and their morphology were assessed by 3 different methods. Dynamic light scattering (DLS) assays showed that isolated vesicles displayed a mean diameter ranging between 43 and 64 nm (Fig. 1A), which was consistent with the exosome population. DLS intensity profiles showed that the major exosome population was approximately 30–50 nm in diameter and a second minor population was approximately 130–150 nm (Fig. 1A). Moreover, samples were visualized using transmission electron microscopy (TEM). Figure 1C shows the presence of small spheres with diameters between 30 and 120 nm in samples obtained with both isolation procedures and with both freezing times (exosomes isolated from fresh plasma followed by freezing or exosomes isolated from frozen plasma). These results were consistent with those obtained with the DLS assay. Finally, Western blot was used to confirm the presence of specific exosome markers, such as CD63 and HSP70. Antibodies bound a double band over 65 kDa for HSP70 and a single band of approximately 45–50 kDa for CD63 (Fig. 1B). The absence of the negative markers GM130 and CytC (data not shown) confirmed the lack of impurities.

### Selection of an exosomal miRNA normalizer

We used a specific primer set to detect each of the miRNAs selected in our set (Table 2). We assessed the sample integrity during RNA extraction, retrotranscription (RT) and RT-qPCR using 4 spike-in controls (data not shown). Hemolysis can affect miRNA stability in free-cell biofluids^28^. Two of the miRNAs studied, miR-23a and miR-451, were used to ensure the non-hemolytic state of samples^28,29^ (Supplementary Table 1). Due to controversy about the normalization of exosomal miRNAs, we used the RefFinder online tool to identify the most stable and best normalizer miRNA^30^. BestKeeper, Normfinder and geNorm software identified let-7a as the most stable miRNA and best control in samples obtained from both isolation methods. This miRNA was used to normalize miRNA levels in further experiments.

**Table 2:** miRNA set studied.

hsa-miR-21-5p LNA PCR primer set, UniRT
hsa-miR-126-5p LNA PCR primer set, UniRT
hsa-miR-16-5p LNA PCR primer set, UniRT
hsa-miR-143-3p LNA PCR primer set, UniRT
hsa-miR-145-5p LNA PCR primer set, UniRT
hsa-miR-150-5p LNA PCR primer set, UniRT
hsa-miR-155-5p LNA PCR primer set, UniRT
hsa-miR-222-3p LNA PCR primer set, UniRT
hsa-miR-320a LNA PCR primer set, UniRT
hsa-let-7 g-5p LNA PCR primer set, UniRT
hsa-miR-630 LNA PCR primer set, UniRT
hsa-let-7a-5p LNA PCR primer set, UniRT

### Effect of freezing before or after exosome isolation

**Figure 2:**
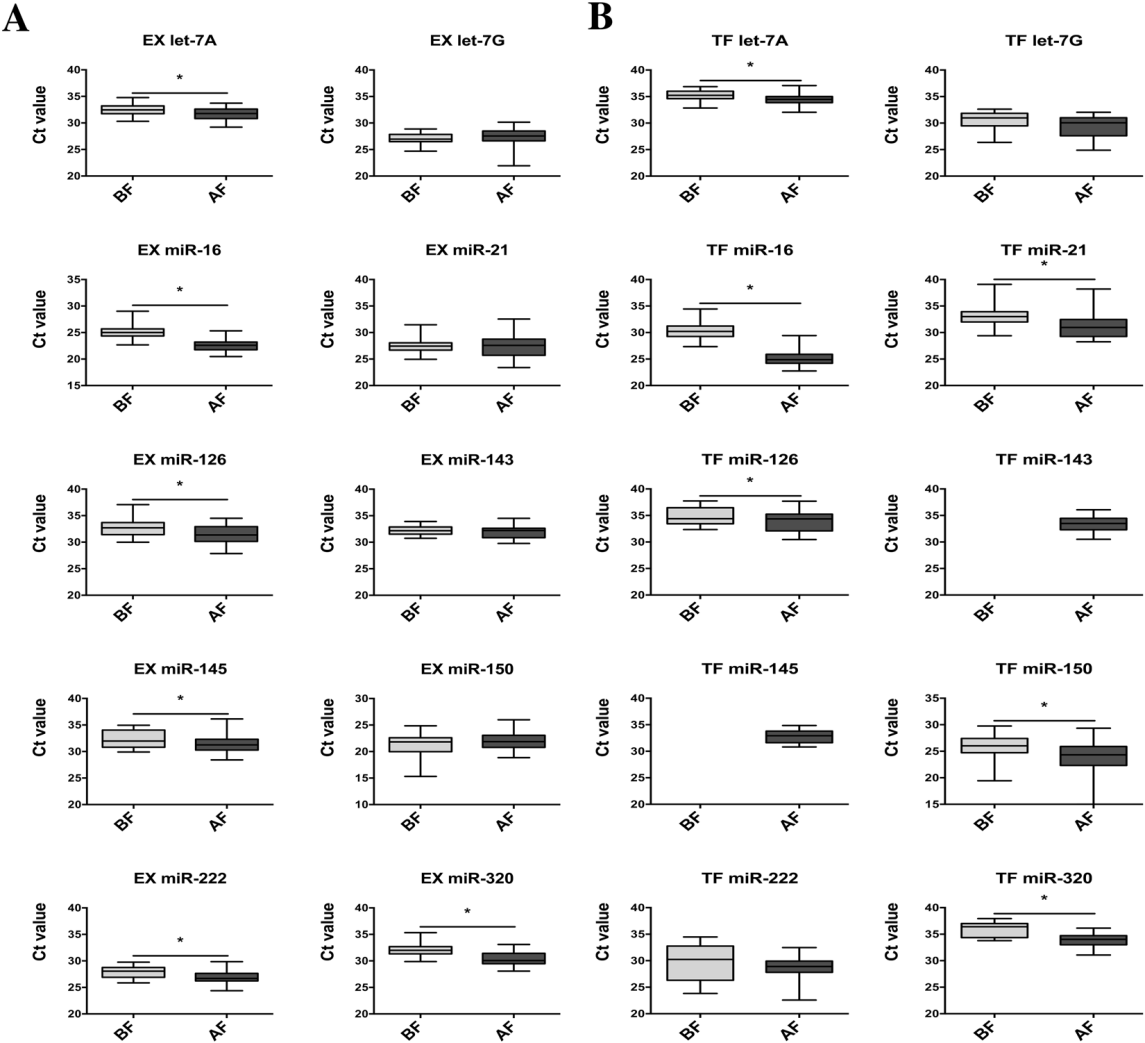
(**A**) Comparison between Ct values of miRNAs obtained from frozen exosomes isolated from fresh plasma (BF) or fresh exosomes isolated from frozen plasma (AF) using the miRCURY Exosome Isolation Kit (Exiqon). (**B**) Comparison between the Ct values of miRNAs obtained from frozen exosomes isolated from fresh plasma (BF) or fresh exosomes isolated from frozen plasma (AF) using the Total Exosome Isolation Kit (Thermo Fisher Scientific). All the data are presented as the mean values (n = 7) (error bars correspond to the standard deviation). *p < 0.05.

Although the morphological stability of exosomes has been previously described^31^, the effect of freezing on exosomal miRNA determination is unclear. In this study, the miRNAs were quantified in exosomes frozen for several weeks after isolation from fresh plasma (“before freezing”, BF) and in exosomes isolated from frozen plasma (“after freezing”, AF) and analyzed immediately after isolation. We evaluated the effect of freezing on the DLS measures and TEM images. The DLS profiles shown in Fig. 1A are consistent with the presence of exosomes in our samples. Non-significant differences were identified between their mean diameters. Nevertheless, major diameter dispersion was observed in exosomes BF. In the TEM images, we visualized the presence of spheres with coherent diameters, as shown in Fig. 1C. When we compared the differences between both freezing cycles used, we observed a major background in exosomes BF. In addition, these samples showed more salt precipitates (images not shown). In general, miRNA cycle threshold (Ct) values were higher (a lower amount of miRNAs) in samples obtained from frozen exosomes using both exosome isolation kits (Fig. 2). Using an EX kit, Ct values for the miRNAs let7a, miR-16, miR-126, miR-145, miR-222 and miR-320 in samples from exosomes isolated from fresh plasma (BF) were significantly higher (lower amount of miRNAs) than the values obtained from AF samples. Similarly, in TF exosomes, the levels of let7a, miR-16, miR-21, miR-126, miR-150 and miR-320 were also significantly lower (higher Ct values) in BF than those in AF samples. In addition, amplification signals for miR-143 and miR-145 were too low for quantification in TF-BF exosomes. Due to the low miRNA levels detected in exosomes isolated by TF-BF, we only evaluated the performance of the other three exosome isolation methods (TF-AF, EX-BF and EX-AF) in further experiments.

### Inter-individual dispersion

Both EX methodologies displayed lower variance distribution (coefficient of variance [CV] < 1) than the TF assay. TF-BF showed low variance of four of the miRNAs analyzed, let-7 g, miR-150, miR-222 and miR-16. For the EX-BF method, most of the miRNAs displayed low variance, including let-7 g, miR-126, miR-143, miR-145, miR-150, miR-320 and miR-16, while only miR-21 and miR-222 displayed high variance (CV > 1). Finally, low variance was observed for EX-AF in six of the miRNAs studied, miR-let-7 g, miR-21, miR-150, miR-222, miR-320 and miR-16.

### Stability of miRNA levels in exosomes over time

**Figure 3:**
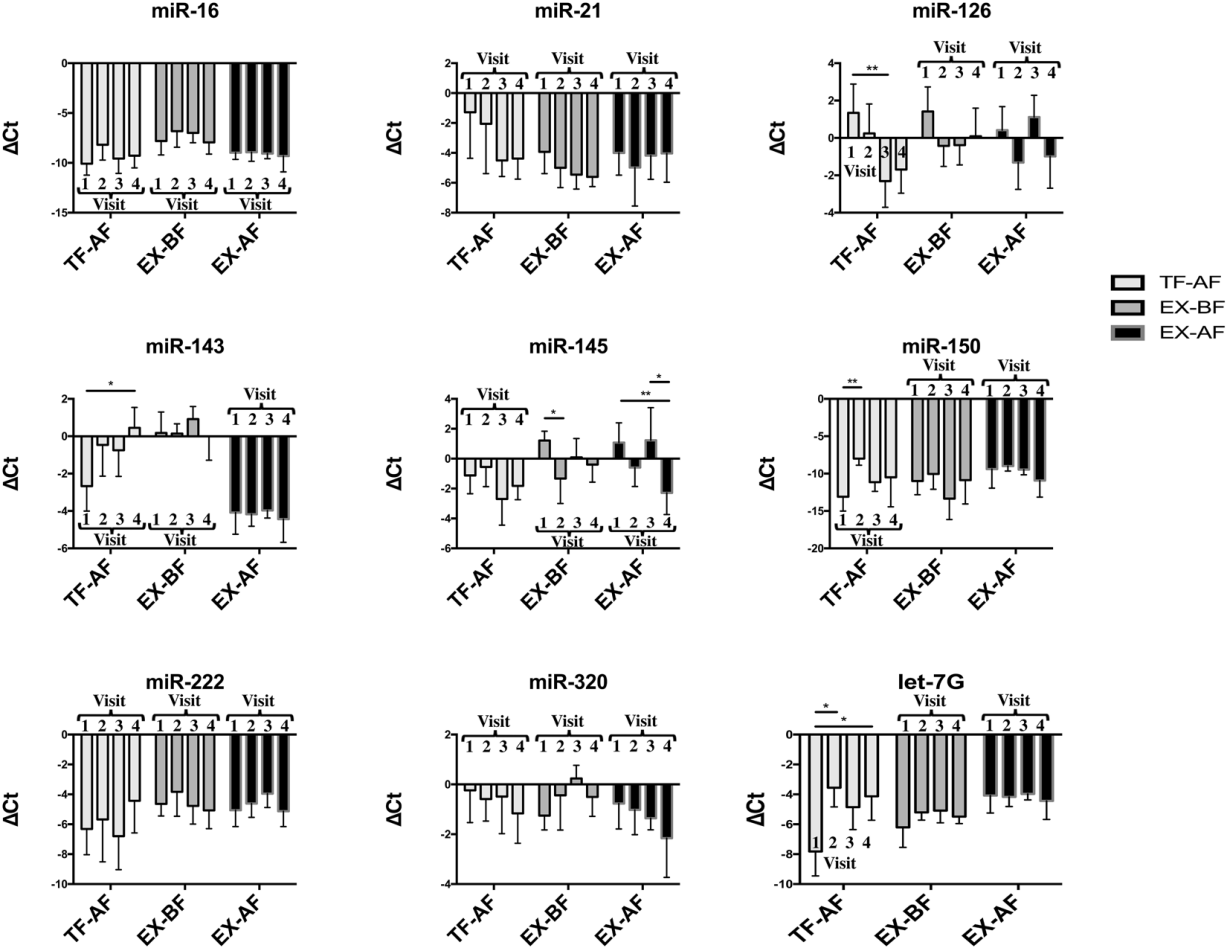
Evaluation of ΔCt (the miRNA Ct value minus the normalizer Ct value, let-7a, of the corresponding time) values over 4 consecutive weeks (V1; V2; V3; and V4) of the miRNA set from exosomes isolated by the three confident methods (TF-AF; EX-BF; and EX-AF). All the data are presented as the mean values (n = 7) (error bars correspond to the standard deviation). *p < 0.05; **p < 0.01.

First, we compared normalized miRNA levels (ΔCt) in exosomes of patients obtained in the four consecutive visits (Fig. 3). In TF-AF exosomes, we observed significant differences for the miRNAs let-7 g, miR-126, miR-143 and miR-150. Specifically, let-7 g was significantly lower in visit 1 (ΔCt = −7.819) than visit 2 (ΔCt = −3.554) (P = 0.043) and visit 4 (ΔCt = −5.984) (P = 0,023); the miR-126 signal was higher in visit 1 (ΔCt = 1,345) than visit 3 (ΔCt = −2.313) (P = 0.0061); miR-143 was significantly higher in visit 4 (ΔCt = 0.451) than visit 1 (ΔCt = −2.672) (P = 0.012) and miR-150 displayed a significant increase in visit 2 (ΔCt = −7.992) compared with visit 1 (ΔCt = −13.100) (P = 0.002). miRNAs from exosomes isolated by EX kits from both BF and AF showed significant differences only for miR-145. The levels of this marker in EX-BF exosomes from visit 1 (ΔCt = 1.211) and visit 2 (ΔCt = −1.330) were significantly different (P = 0.013), while those obtained with EX-AF displayed significant changes between visit 1 (ΔCt = 1.081) and visit 4 (ΔCt = −2.285) (P = 0.0021) and between visit 3 (ΔCt = 1.227) and visit 4 (ΔCt = −2.285) (P = 0.022).

**Figure 4:**
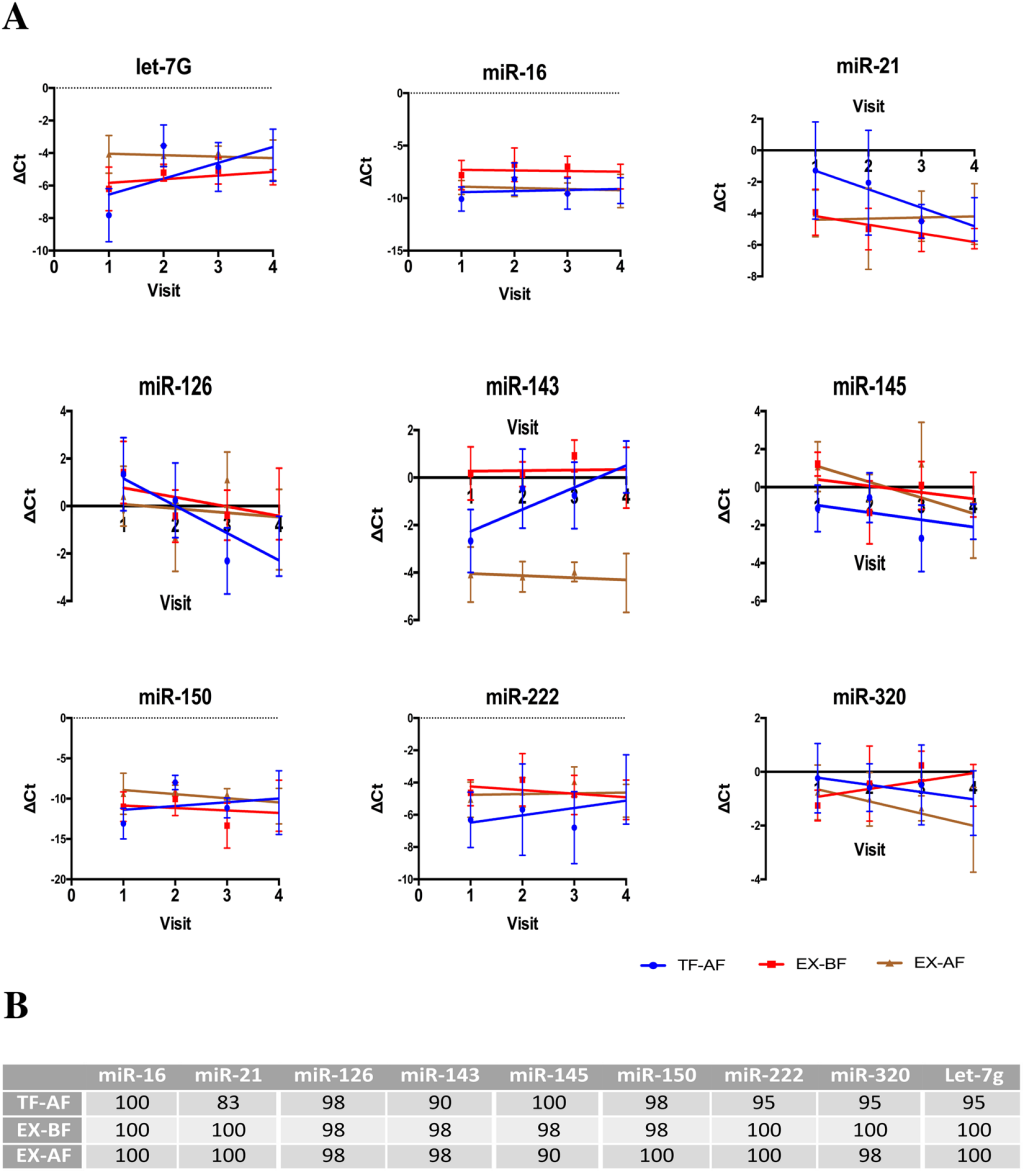
(**A**) Linear regression of ΔCt values between the 4 consecutive visits (V1; V2; V3; and V4) for the three confident methods (TF-AF; EX-BF; and EX-AF). All the data are presented as the mean ± SD. (**B**) The percentage of visit sample differences is included between the confidence limits of the Bland-Altman test.

Then, we performed linear regression analyses of miRNA levels at the four visits, and we estimated the significance of the slope deviation from 0 (Fig. 4A). MiRNA levels in TF-AF exosomes showed slopes significantly different from 0 in four of the miRNAs: let-7 g (P = 0.006), miR-21 (P = 0.0009), miR-126 (P = 0.0002) and miR-143 (P = 0.0008). Levels of miRNAs isolated with the EX-BF and EX-AF methods displayed only one miRNA whose slope was significantly different from 0, miR-21 (P = 0.008) and miR-145 (P = 0.02).

To assess the agreement between the four consecutive measures of each sample, we applied the Bland-Altman test to plot and compare miRNA levels obtained with the three methods selected, TF-AF, EX-BF and EX-AF. Figure 4B shows the percentage of visit sample differences included between the confidence limits. TF-AF had the highest variability, and miR-21 and miR-143 showed the lowest percentages of concordance, 83% and 90% respectively. In contrast, samples obtained with the EX kit, both BF and AF, displayed percentages of concordance over 98% for all miRNAs, except for miR-145, which only showed 90% for EX-BF.

We used the same methodology to compare concordance between measures performed BF and AF with the EX kit. The percentage of differences between both methodologies within 95% confidence limits was over 90% for all miRNAs studied.

Finally, we evaluated the CV between the four visits in each subject. Table 3 shows the average CV for each miRNA and isolation method. TF-AF and EX-BF exosomes displayed higher variability than those of EX-AF, with the CV higher than 1 for four of the miRNAs studied. In the case of TF-AF, these miRNAs were miR-21, miR-126, miR-143 and miR-320, while for EX-BF, they were miR-126, miR-143, miR-145 and miR-320. EX-AF generally showed low CV values, with CV higher than 1 just in two miRNAs, miR-126 and miR-145.

**Table 3:** Coefficient of variance between the 4 consecutive visits.

	miR-16	miR-21	miR-126	miR-143	miR-145	miR-150	miR-222	miR-320	miR-let 7 g
TF-AF	0,14 ± 0,05	1,54 ± 2,34	9,7 ± 16	4,51 ± 5,74	0,81 ± 0,45	0,25 ± 0,07	0,36 ± 0,3	2,36 ± 1,70	0,44 ± 0,11
EX-BF	0,14 ± 0,08	0,24 ± 0,12	8,3 ± 11	4,18 ± 3,25	2,59 ± 1,70	0,19 ± 0,07	0,23 ± 0,12	3,25 ± 2,69	0,17 ± 0,05
EX-AF	0,09 ± 0,04	0,40 ± 0,21	2 ± 1,07	0,17 ± 0,08	5,82 ± 3,72	0,10 ± 0,07	0,16 ± 0,04	0,63 ± 0,35	0,17 ± 0,08

*Abbreviation*. AF: after freezing, BF: before freezing.

## Discussion

To the best of our knowledge, this is the first study evaluating intra-individual and inter-individual variability of miRNAs from circulating exosomes. We found that most of the miRNAs studied showed intra- and inter-individual stability when the miRNA signal in the qPCR amplification was sufficient (Ct < 35).

Research on exosomes has increased exponentially during the last 10 years^32^. Currently, their role in biological functions such as cell-to-cell communication or immune response is known^2^. However, although they participate in the progression of various pathologies, such as cancer^33–35^ or cardiovascular diseases^36^, their specific role and the underlying mechanisms in these pathologies are still unknown. Numerous investigations have focused on their cargos, and multiple studies have described their protein, lipid and RNA cargos^37^. Nevertheless, each new study describes new particles in their cargo, indicating that the exosomes remain unknown.

Some studies have assessed the specificity of exosome cargo depending on the pathological state and the type of cells^38–40^. Their presence in readily accessible biofluids has made exosomes and their specific cargo potential biomarkers. In fact, research on cancer and cardiovascular diseases has suggested that some exosomes’ cargos including miRNAs, lipids and proteins can provide unique opportunities for biomarker discovery and the development of noninvasive diagnosic tools^41–43^. Despite these discoveries, their clinical use remains rare.

One of the major barriers to routine diagnostic use of exosomes is the methodology applied for their isolation. Thus far, there is not a gold standard for exosome isolation^44,45^. The first method proposed as a reference was ultracentrifugation. In early studies, it was shown to be reproducible and provided optimal amounts of exosomes^2,46,47^. However, further analyses have identified some disadvantages in this method, including excessive pressure suffered by exosomes during this process, lack of specificity during the precipitation, excessive time, the equipment required for isolation, and difficulties in exactly reproducing the isolation in different places^48^. Another isolation method commonly used is size exclusion chromatography. It allows a better degree of purity and is less harmful to exosomes^49^. Nevertheless, the high final dilution of the exosome sample makes it difficult to use them in downstream applications that require a high exosome concentration, such as the evaluation of their miRNA profile. Finally, during recent years, there has been an increase in the number of commercial kits developed for exosome isolation. Most of them are based on precipitation. Although they are not completely specific and precipitate some impurities, their rapidity and reproducibility even in different labs make them useful for future diagnosis, primarily in miRNA-based tests.

For implementation of the use of new biomarkers into clinical practice, the first step is to standardize their measurement and to evaluate their stability. The miRNA levels obtained from exosomes isolated from fresh plasma that were subsequently frozen (BF) displayed significantly higher Ct values (lower miRNA levels) for both kits, although it was impossible to quantify some miRNAs in TF-BF exosomes. These findings indicated the importance of the freezing cycle and the time when it is performed. According to our results, it would be more appropriate to isolate exosomes from frozen plasma and avoid further freezing than to isolate them from fresh plasma and preserve them by freezing. Although freezing the exosomes did not significantly alter their morphology, more impurities and background were apparent, which can damage part of the exosomes and complicate their downstream applications. Increasing impurities can lead to the breakage of some exosomes, causing decreased miRNAs levels as observed in this study. In addition, the presence of abundant background can affect the efficiency of miRNA isolation, retrotranscription and RT-qPCR, accounting for the changes observed in miRNAs levels. When using frozen plasma, the levels of miRNAs were higher in exosomes obtained with the EX kit than those from the other kit. Although both kits (TF and EX) allowed miRNA quantification, the differences observed could be explained by a better compatibility of downstream applications between kits. In fact, the total RNA isolation procedure using Exiqon LNA technology, which is the method used in this work, recommends the miRCURY Exosome Isolation Kit for the extraction. The other analyses confirmed the similarity of the 3 isolation methodologies used to assess patients during the 4 weeks, as they showed only subtle variations in specific measurements.

In conclusion, the commercial kits used in this study provided sufficient levels of exosomes to obtain a correct amplification signal for miRNA quantification using RT-qPCR. This study demonstrated the importance of freezing plasma before exosome isolation, RNA isolation and qPCR for miRNAs rather than freezing exosomes before miRNA analysis. Finally, although this work analyzed a small number of miRNAs in a subset of controls, it is the first step to using exosomal miRNAs as future diagnostic markers. Future studies will include a greater variety of RNA types analyzed, including lncRNAs and other miRNAs. Determining the inter- and intra-individual variability of healthy subjects could help to optimize sample size in future studies with circulating exosomes.

## Methods

### Ethics

All the methods were carried out in accordance with relevant guidelines and regulations. All the experimental protocols were approved by the Instituto Investigación Sanitaria Aragón Institutional Review Board (CEICA number 15/2016). In accordance with the recommendations of the Declaration of Human Rights, the Conference of Helsinki and local institutional regulation, written informed consent was obtained from all subjects before the blood extraction.

### Subjects

We recruited healthy volunteers who were non-smokers, did not consume alcohol or any medications and had no history of any chronic medical condition.

### Sample collection

Whole blood samples were drawn using a 21G butterfly needle into EDTA BD Vacutainer blood collection tubes and were then centrifuged at 3,000 g at 4 °C for 15 min. Then, 7 plasma aliquots of 600 μl were taken, two of them were immediately used for exosome isolation, and the rest of the sample was stored at −80 °C.

### Exosome isolation

Two plasma exosome isolation methods were used: miRCURY Exosome Isolation Kit and Total Exosome Isolation Kit. Isolations before and after plasma freezing followed the same methodology. Briefly, for the EX kit, we centrifuged 600 μl of plasma for 5 min at 10,000 g, discarding the pellet. After, we added 6 μl of thrombin to the supernatants and mixed and incubated the samples at room temperature (RT) for 5 min. Samples were then centrifuged with same conditions, and 500 μl from the supernatant was taken for exosome extraction. Then, 200 μl of precipitation buffer was added to the sample, vortexed and incubated at 4 °C for one hour. Samples were centrifuged twice, 5 min at 500 g, before completely removing the supernatant. Finally, pellets were resuspended in 270 μl of PBS and vortexed.

For the TF kit, due to its requirements for the amount of starting sample and for comparison of the results with those obtained with EX kits, we needed to perform 3 isolations from the same sample to reach the 500 μl used in EX kit. The protocol for an individual isolation was as follows: 200 μl of plasma was centrifuged twice at 10,000 g at 22 °C for 22 min, with 180 μl of the supernatant collected after the first centrifugation and 167 μl after the second one. PBS (84 μl) was added to the 167 μl of plasma. Samples were incubated with 7.5 μl of proteinase K at 37 °C during 12 min. Then, 50 μl of precipitation buffer was added and incubated immediately for 35 min at 4 °C. After centrifugation for 6 min at 10,000 g and 22 °C, the supernatant was completely removed, and the pellet was resuspended in 90 μl of PBS. After isolation, 270 μl of exosome suspensions from both kits were treated similarity. Exosomes obtained from fresh plasma were stored at −20 °C until use, while exosomes from frozen plasma were processed for total RNA purification immediately after their isolation.

### Transmission electron microscopy (TEM)

Exosome morphology was evaluated by TEM using the methodology previously described by Lötvall *et al*.^50^. First, total protein in each sample was evaluated by BCA (B9643, C2284, Sigma). A drop of 10 μg of exosomal protein was placed on a Parafilm layer. Exosomes were fixed in 2.5% glutaraldehyde and then washed with deionized water. Samples were contrasted with 2% uranyl acetate, embedded in 0.13% methyl cellulose and 0.4% uranyl acetate and then visualized using a Tecnai T20 microscope (FEI Company), with a filament of LaB_6_. The voltage used during the visualization was 200 KV, and acquiring images was performed with a CCD 2 K × 2 K Veleta model (Olympus).

### Dynamic light scattering (DLS)

The size distribution of nanoparticles was evaluated using DLS assays. An aliquot of 25 µL was diluted in PBS to a final volume of 500 µL and measured in the NanoBrook 90Plus PALS Particle Analyzer (Brookhaven Instruments Corporation). Samples were hit by a diode laser of 35 mW, which allowed discrimination of particle sizes between 0.3 nm and 6 µm.

### Western blot

Western blot analysis was performed to detect two positive markers (CD63 and HSP70) and to confirm the absence of two negative markers (GM130 and CytC). We first performed a BCA protein assay (B9643, C2284, Sigma) to quantify total protein in the samples. In each Western blot assay, after denaturation, 20 μg of total protein was loaded into an ExpressPlus PAGE Gel (GenScript). Proteins were transferred onto a PVDF membrane, Hybond-P (GE Healthcare Life Sciences). Incubation with primary antibodies (anti-CD63 rabbit IgG EXOAB-CD63A-1 [System Bioscience], anti-HSP70 mouse-monoclonal IgG sc32239 [Santa Cruz Biotechnology], anti-cytC mouse-monoclonal IgG sc-13156 [Santa Cruz Biotechnology] and anti-GM130 mouse-monoclonal IgG sc-55591 [Santa Cruz Biotechnology]) was carried overnight at 4 °C; all antibodies were diluted 1:1000 in 5% dry milk TBS-T. We diluted the secondary antibodies 1:5000 (goat anti-mouse IgG-HRP sc-2005 [Santa Cruz Biotechnology] and goat anti-rabbit IgG-HRP secondary antibody EXOAB-HRP [System Bioscience]), and they were incubated at RT for 1 hour. Finally, signals were detected with the chemiluminescence system Luminata Crescendo Western HRP Substrate (Millipore) and visualized using a Versadoc Imaging System (Bio-Rad).

### miRNA isolation and quantification

Exosomes obtained from all methodologies were similarly processed. All the processes were carried out in an RNase-free area, using RNase-free tubes and following the necessary measures to avoid miRNA degradation. Total exosomal RNA extraction was carried out using miRCURY RNA Exosome Isolation Kit (Exiqon) following the manufacturer’s recommendations. First, 1 µL of spike-in mix (UniSp2, UniSp4 and UniSp5) was added to 60 µL of lysis solution, mixed with 200 µL of exosome solution and incubated at RT for 3 min. Then, 20 µL of protein precipitation buffer was added, vortexed and incubated for 1 min at RT. Samples were centrifuged for 3 min at 11,000 g before collecting the supernatant and adding 400 µL of isopropanol. This solution was loaded onto the microRNA Mini Spin Column BF columns. Different cycles of washing were performed until the final elution of RNA. To recover total RNA, we used 100 µL RNase-free H_2_O. Total RNA was stored at −70 °C. The miRCURY LNA Universal RT microRNA PCR kit (Exiqon) was used for reverse transcription. Before the RT reaction, 0.5 µL of another spike-in mix (UniSp6 and cel-miR-39) was added. cDNA was stored at −20 °C until use. Just before using, cDNA was diluted 1:80 in nuclease-free H_2_O. Finally, RT-qPCR was performed using 4 µL of diluted cDNA, 5 µL of PCR Master Mix (Exiqon) and 1 µL of LNA PCR Primer Mix. In addition to the primer set analyzed, 4 primers corresponding to UniSp2, UniSp4, UniSp5 and cel-miR-39 were used to assess sample integrity during RNA extraction, RT and RT-qPCR. The PCR reaction was run in an Applied Biosystems ViiA 7 system using the recommended manufactured protocol.

### Statistical analysis

CV was calculated to assess the inter-individual variability of the ΔCt values of miRNA, and linear regression was performed to assess intra-individual stability in successive blood extractions. Bland-Altman tests were used to evaluate the consistency of the miRNA cargo in exosomes obtained with two different isolation kits. Finally, to select the best normalizer, we used a RefFinder web-based comprehensive tool^30^. This program compares the ranking obtained with the programs geNorm, Normfinder and BestKeeper and the delta Ct method. It assigns an appropriate weight to an individual miRNA and calculates the geometric mean of their weights, giving an overall comprehensive ranking^30^. Statistical analyses were carried out using GraphPad Prism 6 (GraphPad Software) and SPSS version 23.0 (IBM).

### Data availability statement

The data analyzed during the current study are available from the corresponding author.

## Electronic supplementary material

Supplementary Information

## References

[1] Das S, Halushka MK (2015). Extracellular vesicle microRNA transfer in cardiovascular disease. Cardiovascular pathology: the official journal of the Society for Cardiovascular Pathology.

[2] Admyre C (2007). Exosomes with immune modulatory features are present in human breast milk. Journal of immunology.

[3] Cosme J, Liu PP, Gramolini AO (2013). The cardiovascular exosome: current perspectives and potential. Proteomics.

[4] Raposo G (1996). B lymphocytes secrete antigen-presenting vesicles. The Journal of experimental medicine.

[5] Zitvogel L (1998). Eradication of established murine tumors using a novel cell-free vaccine: dendritic cell-derived exosomes. Nature medicine.

[6] Peters PJ (1989). Molecules relevant for T cell-target cell interaction are present in cytolytic granules of human T lymphocytes. European journal of immunology.

[7] Heijnen HF, Schiel AE, Fijnheer R, Geuze HJ, Sixma JJ (1999). Activated platelets release two types of membrane vesicles: microvesicles by surface shedding and exosomes derived from exocytosis of multivesicular bodies and alpha-granules. Blood.

[8] Wolfers J (2001). Tumor-derived exosomes are a source of shared tumor rejection antigens for CTL cross-priming. Nature medicine.

[9] Caby MP, Lankar D, Vincendeau-Scherrer C, Raposo G, Bonnerot C (2005). Exosomal-like vesicles are present in human blood plasma. International immunology.

[10] Lasser C (2011). Human saliva, plasma and breast milk exosomes contain RNA: uptake by macrophages. Journal of translational medicine.

[11] Hoorn EJ (2005). Prospects for urinary proteomics: exosomes as a source of urinary biomarkers. Nephrology.

[12] Pisitkun T, Shen RF, Knepper MA (2004). Identification and proteomic profiling of exosomes in human urine. Proceedings of the National Academy of Sciences of the United States of America.

[13] Witwer, K. W. *et al*. Standardization of sample collection, isolation and analysis methods in extracellular vesicle research. *Journal of extracellular vesicles* **2**, 10.3402/jev.v2i0.20360 (2013). 10.3402/jev.v2i0.20360 PMC3760646 24009894

[14] Saman S (2012). Exosome-associated tau is secreted in tauopathy models and is selectively phosphorylated in cerebrospinal fluid in early Alzheimer disease. The Journal of biological chemistry.

[15] Kosaka N (2010). Secretory mechanisms and intercellular transfer of microRNAs in living cells. J Biol Chem.

[16] Croce CM (2009). Causes and consequences of microRNA dysregulation in cancer. Nat Rev Genet.

[17] Min PK, Chan SY (2015). The biology of circulating microRNAs in cardiovascular disease. European journal of clinical investigation.

[18] Ferracin M (2015). Absolute quantification of cell-free microRNAs in cancer patients. Oncotarget.

[19] Mangolini A (2015). Diagnostic and prognostic microRNAs in the serum of breast cancer patients measured by droplet digital PCR. Biomarker research.

[20] Hou J, Meng F, Chan LW, Cho WC, Wong SC (2016). Circulating Plasma MicroRNAs As Diagnostic Markers for NSCLC. Frontiers in genetics.

[21] Arroyo JD (2011). Argonaute2 complexes carry a population of circulating microRNAs independent of vesicles in human plasma. Proceedings of the National Academy of Sciences of the United States of America.

[22] Vickers KC, Palmisano BT, Shoucri BM, Shamburek RD, Remaley AT (2011). MicroRNAs are transported in plasma and delivered to recipient cells by high-density lipoproteins. Nature cell biology.

[23] Valadi H (2007). Exosome-mediated transfer of mRNAs and microRNAs is a novel mechanism of genetic exchange between cells. Nature cell biology.

[24] Kondkar AA, Abu-Amero KK (2015). Utility of circulating microRNAs as clinical biomarkers for cardiovascular diseases. BioMed research international.

[25] Shurtleff, M. J., Temoche-Diaz, M. M., Karfilis, K. V., Ri, S. & Schekman, R. Y-box protein 1 is required to sort microRNAs into exosomes in cells and in a cell-free reaction. *Elife* **5**, 10.7554/eLife.19276 (2016). 10.7554/eLife.19276 PMC5047747 27559612

[26] Villarroya-Beltri C (2013). Sumoylated hnRNPA2B1 controls the sorting of miRNAs into exosomes through binding to specific motifs. Nat Commun.

[27] Rabinowits G, Gercel-Taylor C, Day JM, Taylor DD, Kloecker GH (2009). Exosomal microRNA: a diagnostic marker for lung cancer. Clinical lung cancer.

[28] Kirschner MB (2013). The Impact of Hemolysis on Cell-Free microRNA Biomarkers. Front Genet.

[29] Shah JS, Soon PS, Marsh DJ (2016). Comparison of Methodologies to Detect Low Levels of Hemolysis in Serum for Accurate Assessment of Serum microRNAs. PLoS One.

[30] Xie, F., Xiao, P., Chen, D., Xu, L. & Zhang, B. miRDeepFinder: a miRNA analysis tool for deep sequencing of plant small RNAs. *Plant molecular biology*, 10.1007/s11103-012-9885-2 (2012). 10.1007/s11103-012-9885-2 22290409

[31] Laulagnier K (2004). Mast cell- and dendritic cell-derived exosomes display a specific lipid composition and an unusual membrane organization. Biochem J.

[32] Schey KL, Luther JM, Rose KL (2015). Proteomics characterization of exosome cargo. Methods.

[33] Boyiadzis, M. & Whiteside, T. L. The emerging roles of tumor-derived exosomes in hematological malignancies. *Leukemia*, 10.1038/leu.2017.91 (2017). 10.1038/leu.2017.91 28321122

[34] Javeed, N. & Mukhopadhyay, D. Exosomes and their role in the micro-/macro-environment: a comprehensive review. *J Biomed Res* **30**, 10.7555/JBR.30.20150162 (2016). 10.7555/JBR.30.20150162 PMC5706431 28290182

[35] Steinbichler, T. B., Dudas, J., Riechelmann, H. & Skvortsova, II. The Role of Exosomes in Cancer Metastasis. *Semin Cancer Biol*, 10.1016/j.semcancer.2017.02.006 (2017). 10.1016/j.semcancer.2017.02.006 28215970

[36] Boulanger CM, Loyer X, Rautou PE, Amabile N (2017). Extracellular vesicles in coronary artery disease. Nat Rev Cardiol.

[37] Keerthikumar S (2016). ExoCarta: A Web-Based Compendium of Exosomal Cargo. J Mol Biol.

[38] Goetzl, E. J. *et al*. Altered cargo proteins of human plasma endothelial cell-derived exosomes in atherosclerotic cerebrovascular disease. *FASEB J*, 10.1096/fj.201700149 (2017). 10.1096/fj.201700149 PMC5503715 28476896

[39] Soung, Y. H., Ford, S., Zhang, V. & Chung, J. Exosomes in Cancer Diagnostics. *Cancers (Basel)* **9**, 10.3390/cancers9010008 (2017). 10.3390/cancers9010008 PMC5295779 28085080

[40] Diaz-Hidalgo L (2016). Transglutaminase type 2-dependent selective recruitment of proteins into exosomes under stressful cellular conditions. Biochim Biophys Acta.

[41] Thind A, Wilson C (2016). Exosomal miRNAs as cancer biomarkers and therapeutic targets. J Extracell Vesicles.

[42] Nuzhat Z (2017). Tumour-derived exosomes as a signature of pancreatic cancer - liquid biopsies as indicators of tumour progression. Oncotarget.

[43] Chistiakov, D. A., Orekhov, A. N. & Bobryshev, Y. V. Cardiac Extracellular Vesicles in Normal and Infarcted Heart. *Int J Mol Sci* **17**, 10.3390/ijms17010063 (2016). 10.3390/ijms17010063 PMC4730308 26742038

[44] Mateescu B (2017). Obstacles and opportunities in the functional analysis of extracellular vesicle RNA - an ISEV position paper. J Extracell Vesicles.

[45] Taylor DD, Shah S (2015). Methods of isolating extracellular vesicles impact down-stream analyses of their cargoes. Methods.

[46] Mathias RA, Lim JW, Ji H, Simpson RJ (2009). Isolation of extracellular membranous vesicles for proteomic analysis. Methods Mol Biol.

[47] Fernandez-Llama P (2010). Tamm-Horsfall protein and urinary exosome isolation. Kidney Int.

[48] Li P, Kaslan M, Lee SH, Yao J, Gao Z (2017). Progress in Exosome Isolation Techniques. Theranostics.

[49] Mol, E. A., Goumans, M. J., Doevendans, P. A., Sluijter, J. P. & Vader, P. Higher functionality of extracellular vesicles isolated using size-exclusion chromatography compared to ultracentrifugation. *Nanomedicine*, 10.1016/j.nano.2017.03.011 (2017). 10.1016/j.nano.2017.03.011 28365418

[50] Lasser, C., Eldh, M. & Lotvall, J. Isolation and characterization of RNA-containing exosomes. *Journal of visualized experiments: JoVE*, e3037, 10.3791/3037 (2012). 10.3791/3037 PMC3369768 22257828

