# Supplementary material for: Stability of Circulating Exosomal miRNAs in Healthy Subjects
Source: Sci Rep. 2018 Jul 9;8:10306. doi: 10.1038/s41598-018-28748-5 (PMC6037782; doi:10.1038/s41598-018-28748-5)
Supplement: Supplementary file 1 — Supplementary Information [file 41598_2018_28748_MOESM1_ESM.doc]

Stability of Circulating Exosomal miRNAs in Healthy Subjects

David Sanz-Rubio, MS, Inmaculada Martin-Burriel, PhD, Ana Gil, RN, Pablo Cubero, RN, Marta Forner, RN, Abdelnaby Khalyfa, PhD, Jose M. Marin, MD.


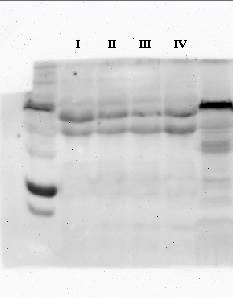


Supplementary Figure 1. Complete image from the western blot of HSP70 with samples corresponding to I) TF-AF, II) TF-BF, III) EX-AF and IV) EX-BF.


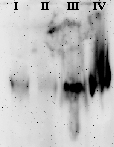


Supplementary Figure 2. Complete image from the western blot of CD63 with samples corresponding to I) TF-AF, II) TF-BF, III) EX-AF and IV) EX-BF.

Supplementary Table 1. The coefficient of hemolysis for each sample subject at the four isolation times. Coefficients of hemolysis were calculated as the miR-451a Ct: the miR23a Ct. (a ratio<5 is considered a low hemolytic risk^1^).

| Hemolysis Coefficient | | | | |
| --- | --- | --- | --- | --- |
| Subjects | V1 | V2 | V3 | V4 |
| S1 | 3,87 | 3,93 | 4,10 | 4,25 |
| S2 | 4,15 | 4,14 | 3,83 | 3,51 |
| S3 | 4,04 | 2,71 | 4,11 | 4,23 |
| S4 | 3,60 | 3,90 | 4,43 | 4,27 |
| S5 | 1,97 | 3,15 | 4,25 | 2,92 |
| S6 | 3,95 | 3,69 | 3,36 | 3,20 |
| S7 | 3,13 | 3,31 | 2,99 | 4,34 |

**References**

1 Shah, J. S., Soon, P. S. & Marsh, D. J. Comparison of Methodologies to Detect Low Levels of Hemolysis in Serum for Accurate Assessment of Serum microRNAs*. PLoS O*n**e** 11, e0153200, doi:10.1371/journal.pone.0153200 (2016).
